# Association of cardiac troponin T and growth differentiation factor 15 with replacement and interstitial cardiac fibrosis in community dwelling adults: The multi-ethnic study of atherosclerosis

**DOI:** 10.3389/fcvm.2023.1104715

**Published:** 2023-02-09

**Authors:** Christopher R. deFilippi, Henry Tran, Raghav Gattani, Lori B. Daniels, Palak Shah, Leonard Ilkhanoff, Robert Christenson, Joao A. Lima, Stephen Seliger

**Affiliations:** ^1^Inova Heart and Vascular Institute, Falls Church, VA, United States; ^2^Division of Cardiology, University of California and San Diego Medical Center, San Diego, CA, United States; ^3^Department of Pathology, University of Maryland School of Medicine, Baltimore, MD, United States; ^4^The Johns Hopkins School of Medicine, Baltimore, MD, United States

**Keywords:** biomarkers, troponin, GDF-15, fibrosis, cardiac disease, heart failure

## Abstract

**Background:**

Subclinical abnormalities in myocardial structure (stage B heart failure) may be identified by cardiac and non-organ specific biomarkers. The associations of high-sensitivity cardiac troponin T (hs-cTnT) and growth differentiation factor-15 (GDF-15) with cardiac magnetic resonance imaging (CMR) interstitial fibrosis (extracellular volume [ECV]) is unknown and for GDF-15 the association with replacement (late gadolinium enhancement [LGE]) is also unknown. GDF-15 is a systemic biomarker also released by myocytes associated with fibrosis and inflammation. We sought to define the associations of hs-cTnT and GDF-15 with these CMR fibrosis measures in the MESA cohort.

**Methods:**

We measured hs-cTnT and GDF-15 in MESA participants free of cardiovascular disease at exam 5. CMR measurements were complete in 1737 for LGE and 1258 for ECV assessment. We estimated the association of each biomarker with LGE and increased ECV (4th quartile) using logistic regression, adjusted for demographics and risk factors.

**Results:**

Mean age of the participants was 68 ± 9 years. Unadjusted, both biomarkers were associated with LGE, but after adjustment only hs-cTnT concentrations remained significant (4th vs. 1st quartile OR] 7.5, 95% CI: 2.1, 26.6). For interstitial fibrosis both biomarkers were associated with 4th quartile ECV, but the association was attenuated compared to replacement fibrosis. After adjustment, only hs-cTnT concentrations remained significant (1st to 4th quartile OR 1.7, 95%CI: 1.1, 2.8).

**Conclusion:**

Our findings identify that both interstitial and replacement fibrosis are associated with myocyte cell death/injury, but GDF-15 a non-organ specific biomarker prognostic for incident cardiovascular disease is not associated with preclinical evidence of cardiac fibrosis.

## Introduction

Heart failure (HF) continues to be a major source of morbidity and mortality with a reported rise in prevalence, hospitalization, and mortality (1). Although studies have reported a decrease in incidence of HF with reduced ejection fraction (HFrEF), the rising burden of HF with preserved ejection fraction (HFpEF), often the result of a confluence of systemic comorbidities, continues to challenge HF management despite recent successes with medical treatment and physical activity (2–4).

Circulating biomarkers representing cardiac injury and strain; systemic processes such as inflammation; and renal dysfunction are associated with an increased risk of incident HF (5–8). Identifying a preclinical phenotype using cardiac magnetic resonance (CMR), amino terminal B-type natriuretic peptide (NT-proBNP) was associated with interstitial (diffuse) fibrosis represented by an increase in extracellular volume (ECV) and high sensitivity cardiac troponin T (hs-cTnT) was associated with the presence and extent of replacement (focal) fibrosis (7, 9). Elevations of these biomarkers characterize stage B or pre-HF, defined by the presence of myocardial abnormalities in the absence of symptoms (10). Given that progression to HFpEF often represents an insidious systemic process associated with inflammation and fibrosis, we sought to study growth differentiation factor-15 (GDF-15), a singular powerful prognostic marker associated with incident HF and death in the general population and with HF hospitalization and death in patients with symptomatic HFpEF (11–14). GDF-15 is a non-cardiac-specific circulating cytokine expressed in various tissues, including macrophages, smooth muscle, endothelium and myocytes (15). It is considered a marker of oxidative stress, endothelial dysfunction inflammation and potentially fibrosis (13, 15). It has been also classified as an intermediary biomarker representing the confluence of multiple systemic processes (14). Evolution of CMR to identify surrogates of both replacement and interstitial cardiac fibrosis can provide mechanistic insights into the pre-clinical pathology reflected by these two circulating prognostic biomarkers and potentially point to opportunities for early interventions (16).

Given the strength of the prognostic association of GDF-15 with incident HF we hypothesized progressive concentrations would be associated with cardiac fibrosis in persons without known CVD. Using a cross-sectional study design, we aimed to quantify the association of GDF-15 with CMR evidence of both replacement and interstitial fibrosis in community dwelling individuals without known cardiovascular disease (CVD) within the Multi-Ethnic Study of Atherosclerosis (MESA) cohort and contrast this to hs-cTnT.

## Materials and methods

### Study population

MESA was initiated in July 2000 to investigate the prevalence, correlates, and progression of subclinical CVD in a population-based sample of 6,814 men and women aged 45-84 years and of 4 self-reported ethnicities (non-Hispanic white, black, Hispanic, and Chinese). The participants enrolled were free of known CVD and were extensively evaluated with questionnaires, physical examination, laboratory tests, and imaging upon entry into the study (17). The presence of subclinical CVD was unknown at the time of enrollment. For our study we included all participants attending exam 5 (2010-2012) who had adequate plasma available for the measurement of the two biomarkers, hs-cTnT and GDF-15. Among these, the total number of participants with completed CMR was 3,015. Of those, the 1,763 who underwent gadolinium enhancement CMR, and who did not have an interim CVD event between study inception and the exam 5 CMR comprised our study cohort. All participants provided informed consent for participation. MESA was approved by the Institutional Review Boards of the University of Washington and the participating sites; the measurement of hs-cTnT and GDF-15 were approved by the Institutional Review Board of the Inova Health System, Falls Church, Virginia.

### Biomarker assay measurement

Hs-cTnT and GDF-15 were measured from previously frozen EDTA plasma collected at exam 5, on the Cobas e602 (Roche Diagnostics) located at Inova Heart and Vascular Institute, Falls Church, VA. The methodology was consistent with the measurement of hs-cTnT measured at earlier time points in MESA (7). The non-gender specific 99*^th^* percentile reference range for hs-cTnT in the United States has been defined as < 19 ng/L (18). The median GDF-15 in apparently healthy individuals has been reported as 808 ng/L (interquartile range 608-1,308 ng/L) (19).

### CMR imaging and analysis

**FIGURE 1:**
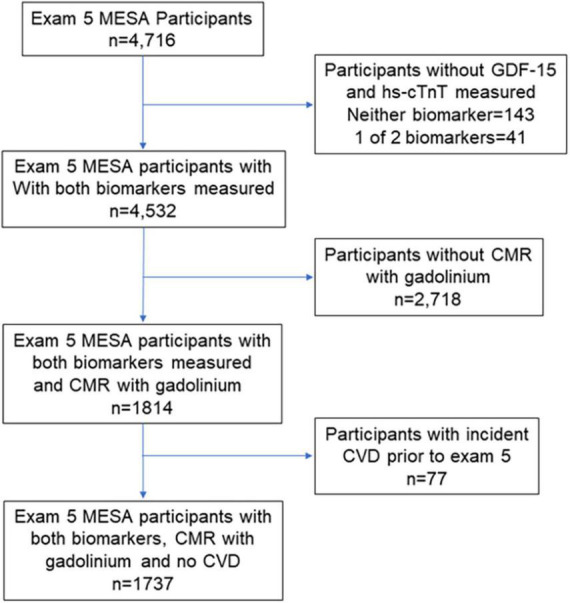
Consort diagram of the Multi-Ethnic Study of Atherosclerosis (MESA) participants at exam 5 (years 2010-2012) who had biomarkers retrospectively measured and cardiac magnetic resonance (CMR) performed with gadolinium.

The CMR at exam 5 has previously been described (20, 21). Contrast enhanced CMR using LGE was performed among those without contraindications to gadolinium and with an estimated glomerular filtration rate ≥ 45 mL/min/1.73 m^2^ (≥ 60 mL/min1.73m^2^ for the site at Northwestern University) were qualified to participate. LGE images were acquired 10 to 15 min after intravenous administration of 0.15 mmol/kg gadolinium–DTPA with breath-held segmented inversion recovery sequence and acquired in the same orientations as the cine images. Inversion times were adjusted to null normal myocardium. Myocardial replacement fibrosis was defined as focal LGE either in 2 adjacent short axis slices or in one short axis and one long axis image at a corresponding location using Qmass (version 7.2, Medis). Figure 1 shows which participants were included in this analysis based on having a measurement of both hs-cTnT and GDF-15, CMR with gadolinium at exam 5, and no clinical incident CVD event since study inception.

For evaluation of interstitial fibrosis, 1 short axis pre-contrast modified look locker inversion recovery image at the mid slice position was acquired, repeated at 12 and 25 min after contrast injection. The timing was chosen to be compatible with previous studies and to accommodate the design of the entire CMR protocol. The protocol is described in detail elsewhere (20). Interstitial myocardial fibrosis was quantified as percentage of total ECV. In MESA at exam 5, hematocrit was measured in only 608 (45.5%) of the participants who underwent T1 mapping. For the remainder, a synthetic hematocrit was calculated that correlated closely with the measured hematocrit and was used to calculate myocardial ECV (20).

### Statistics

Characteristics of participants with complete measures of replacement fibrosis and diffuse fibrosis were described by frequencies for categorical variables and means (standard deviations) or medians (interquartile range [IQR]) for continuous variables, as appropriate. Correlation between GDF-15 and hs-cTnT concentrations were estimated with Spearman rank correlation coefficient. Associations of each biomarker with a) LGE and b) diffuse fibrosis (defined as the highest quartile of ECV) were estimated with logistic regression models. Each biomarker was modeled separately as a continuous variable (after natural log (ln)-transformation due to skewed distributions) and as categorical variables in quartiles of distribution. Models were adjusted for demographic factors (age, gender, race/ethnicity [Black, White, Chinese American, Hispanic]) and cardiovascular risk factors (hypertension, diabetes, lipid levels, smoking, and estimated glomerular filtration rate). In sensitivity analyses, individuals with LGE were excluded from analyses of diffuse fibrosis. For all logistic models, goodness of fit of logistic models was assessed with the Hosmer-Lemeshow test. Associations were presented as odds ratios and 95% confidence intervals. In additional sensitivity analyses, multiple linear regression models were used to estimate the association of each biomarker with%ECV as a continuous measure, adjusted for the same potential confounders described above. All analyses were performed with Stata SE v12.1 (Statacorp, College Station, Texas).

## Results

**FIGURE 2:**
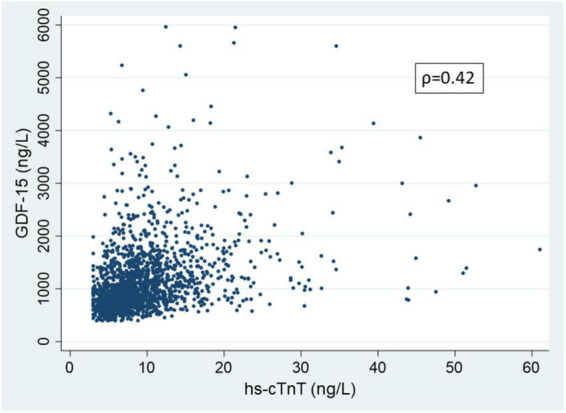
Scatter plot showing the association of growth differentiation factor-15 (GDF-15) and high sensitivity cardiac troponin T (hs-cTnT) in participant undergoing cardiac magnetic resonance and receiving gadolinium at MESA exam 5 (n = 1737). Correlation calculated on entire data; for clarity in presentation, hs-cTnT values greater than 75 ng/L (99.8 percentile) and GDF-15 values greater than 6000 ng/L (99.7 percentile) are not shown.

The baseline characteristics of participants free of known clinical CVD who underwent CMR with Gadolinium at exam 5 (n = 1737) and the subset with measured ECV (n = 1258) are shown in Table 1. Consistent with exam 5 being approximately 10-years after the initiation of MESA, participants were overall in their late 60’s, approximately 49% were women and 25% were black. Of the 1737 participants with CMR with gadolinium, 112 (6.5%) had prevalent focal LGE. For the full gadolinium cohort, median hs-cTnT was 8.1 ng/L (IQR 5.9-11.2 ng/L) and median GDF-15 was 973 ng/L (IQR 738-1348 ng/L). For the cohort with measured ECV median hs-cTnT concentration was 8.0 ng/L (IQR 5.8-11.2 ng/L) and the median GDF-15 concentration was 972 ng/L (IQR 736-1342 ng/L). The median percent ECV for the 4*^th^* quartile versus the lower 3 quartiles was 29.8% (IQR 29.1-30.9%) and 25.7% (IQR 24.3-26.9%) respectively. In Supplementary Tables 1A and 1B, participant baseline characteristics for those with complete LGE measures and the subset with ECV measures respectively are shown across progressive quartiles of GDF-15. Higher concentrations of GDF-15 were associated with more advanced age, a greater prevalence of traditional risk factors for CVD, a lower eGFR, a slightly lower LVEF, but also a lower LV end diastolic volume index lower and in men, a lower LV mass. The trends for progressively higher concentrations of hs-cTnT with demographics and traditional CVD risk factors were similar (Supplementary Tables 2A, B). The correlation between hs-cTnT and GDF-15 was only moderate (ρ = 0.42) in participants who had received gadolinium (*n* = 1737) (Figure 2). In the subgroup with ECV measure the correlation between the two biomarkers was similar (ρ = 0.41).

**TABLE 1:** MESA exam 5 participant characteristics for those with gadolinium enhanced CMR and with ECV measurements.

Baseline characteristics	With contrast-enhanced CMR (*n* = 1737)	With % ECV measured (*n* = 1258)
Age (years)	67.6 (8.8)	67.4 (8.7)
Male	886 (51.0%)	644 (51.2%)
**Race/Ethnicity**		
Caucasian	775 (44.6%)	646 (51.4%)
Chinese American	164 (9.4%)	143 (11.4%)
Black	432 (24.9%)	289 (23.0%)
Hispanic	366 (21.1%)	180 (14.3%)
**Risk factors for CVD**		
HTN	932 (53.7%)	661 (52.5%)
Systolic BP (mm Hg)	122.3 (19.2)	121.5 (19.0)
Diastolic BP (mm hg)	68.9 (9.7)	68.6 (9.7)
Diabetes	278 (16%)	193 (15.3%)
**Smoking**		
Never	754 (43.6%)	540 (43.1%)
Former	833 (48.2%)	612 (48.8%)
Current	143 (8.3%)	102 (8.1%)
LDL-C (mg/dL)	107.1 (31.4)	106.1 (30.8)
HDL-C (mg/d)	54.8 (16.2)	54.3 (15.8)
Triglycerides	110.3 (62.5)	112.5 (65.5)
BMI (kg/m2)	28.4 (5.2)	28.4 (5.3)
eGFR (ml/min/1.73 m^2^)	84.3 (14.4)	84.4 (14.2)
**CMR measures**		
Prevalent LGE	112 (6.5%)	NA
ECV%	NA	26.7 (2.8)
LVEF (%)	61.6 (6.9)	61.9 (6.8)
**LV mass (g)**		
LVEDVI	65.7 (13.5)	65.4 (13.4)
Female	104.7 (21.7)	102.7 (21.1)
Male	145.8 (29.6)	144.4 (29.4)

BP, blood pressure; BMI, body mass index; CMR, cardiac magnetic resonance; eGFR, estimated glomerular filtration rate; ECV, extra cellular volume; HDL-C, high density lipoprotein cholesterol; HTN, Hypertension; LDL-C, low density lipoprotein cholesterol; LVDVI, left ventricular diastolic volume index; LGE, late gadolinium enhancement; LVEF, left ventricular ejection fraction; NA, not applicable.

Progressively higher hs-cTnT concentrations at exam five - whether considered as a continuous variable or by quartiles - were strongly associated with replacement fibrosis as measured by presence of LGE. In an unadjusted model, the odds ratio (OR) for LGE in participants with a hs-cTnT concentration in the 4th versus 1st quartile was 25.1 (95% confidence interval [CI] 7.9-80.7). Even in a highly adjusted model inclusive of demographics, traditional risk factors, and CMR-determined left ventricular mass, subjects with the highest quartile of hs-cTnT had more than 7 times the odds of LGE compared to those with the lowest quartile (adjusted OR = 7.5 [95%CI 2.1-26.6 In contrast, the association of GDF-15 concentration with the presence of LGE was more modest with an unadjusted OR for the 4th to 1st quartile of 3.9 (95%CI 2.7-5.4). After adjusting for demographics, risk factors and left ventricular mass the association of GDF-15 concentration and LGE was no longer significant with a 4th to 1st quartile adjusted odds ratio of 2.0 (95%CI 0.9-4.7). There was likewise no significant association of GDF-15 when considered as a continuous variable with LGE. Details of the unadjusted and adjusted odds ratios for LGE-related replacement fibrosis for both biomarkers are shown in Table 2.

**TABLE 2:** Association (odds ratio) of GDF-15 and hs-cTnT with replacement fibrosis (+ LGE) (*n* = 1737).

	Number with LGE	Unadjusted	Demographic adjusted	Demographic + risk factor + LV mass adjusted
ln GDF-15	112 (6.5%)	2.2 (1.5, 3.2); p < 0.001	1.6 (1.1, 2.5); p = 0.03	1.6 (0.9, 2.7); *p* = 0.1
**GDF-15 quartiles (ng/L)**				
Q1 (< 738)	11 (2.5%)	Ref	Ref	Ref
Q2 (738-973)	24 (5.5%)	2.3 (1.1, 4.7); *p* = 0.03	1.9 (0.9, 4.1); *p* = 0.08	1.6 (0.7, 3.4); *p* = 0.3
Q3 (973-1347)	38 (8.8%)	3.7 (1.9, 7.3); *p* < 0.001	2.8 (1.3, 5.8); *p* = 0.007	2.1 (0.9, 4.6); *p* = 0.06.
Q4 (> 1347)	39 (9.0%)	3.9 (1.9,7.5); *p* < 0.001	2.5 (1.2-5.37); *p* = 0.002	2.0 (0.9, 4.7); *p* = 0.1
ln hs-cTnT	112 (6.5%)	3.8 (2.7, 5.4); *p* < 0.001	2.6 (1.7, 3.8); *p* < 0.001	2.11 (1.3, 3.4); *p* = 0.002
**hs-cTnT quartiles (ng/L)**				
Q1 (< 6)	3 (0.7%)	Ref	Ref	Ref
Q2 (6-8)	17 (3.9%)	5.9 (1.7, 20.1); *p* = 0.005	4.13 (1.2, 14.4); *p* = 0.03	3.3 (1.0, 11.7); *p* = 0.06
Q3 (8-11)	27 (6.2%)	9.5 (2.9, 31.5); *p* < 0.001	5.57 (1.6, 19.0); *p* = 0.006	4.1 (1.2, 14.3); *p* = 0.03
Q4 (11-168)	65 (14.9%)	25.1 (7.9, 80.7); *p* < 0.001	11.8 (3.5, 40.1); *p* < 0.001	7.5 (2.1, 26.6); *p* = 0.002
	Demographics: age, gender, race/ethnicity (Black, White, Chinese American, Hispanic)			
	Risk Factors: Hypertension, diabetes, low- and high-density lipoprotein cholesterol, smoking, estimated glomerular filtration rate			
	Growth differentiation factor-15, GDF-15; High sensitivity cardiac troponin T, hs-cTnT; Late gadolinium enhancement, LGE; Left ventricle, LV; Natural log, ln; Quartile, Q Odds ratios are reported as the OR (95% confidence intervals)			

In contrast to the strong association of hs-cTnT and the presence of replacement fibrosis with LGE, the association of hs-cTnT with interstitial fibrosis (defined as the 4th quartile of CMR-determined ECV) was more modest, with subjects in the 4th quartile of hs-cTnT having a 40% greater odds compared to the lowest quartile (OR = 1.4 [95%CI 1.0-1.9]), an association that was similar after adjustment for demographics and CVD risk factors (OR = 1.7 [95% CI 1.1-2.8]). In an unadjusted model GDF-15 concentrations had a similar association with interstitial fibrosis as hs-cTnT, with an odds ratio of 1.5 (95%CI 1.0-2.1) for the 4th vs. 1st quartile. However, these associations were largely attenuated by adjustment for demographics (adjusted OR 0.9 [95% CI 0.6-1.4]). Results were similar when the biomarkers were modeled as continuous variables, after ln-transformation. Details of the unadjusted and adjusted odds ratios for 4th quartile ECV-related interstitial fibrosis for both biomarkers are shown in Table 3. In sensitivity analyses excluding those participants (*N* = 88) with complete ECV measures who had LGE, the observed associations of hs-cTnT with elevated ECV (per 1-ln unit increment, OR = 1.7 [95%CI 1.2, 2.4]) and of GDF-15 with elevated ECV (per-1ln unit increment, OR = 1.0 [95%CI 0.7, 1.5]) were unchanged. We also evaluated ECV as a continuous variable with linear regression as shown in Supplementary Table 3. Findings were similar to when ECV was dichotomized with values in the 4th quartile defined as ‘elevated’. GDF-15 was significantly associated with ECV only in the unadjusted model whereas hs-cTnT was associated with ECV after adjustment for demographics and comorbidities.

**TABLE 3:** Association (odds ratios) of GDF-15 and hs-cTnT with interstitial fibrosis (highest quartile % ECV) (*n* = 1258).

	% with high ECV	Unadjusted	Demographic adjusted	Demographic + risk factor + LV mass adjusted
ln GDF-15		1.5 (1.1, 1.9); *p* = 0.006	1.1 (0.8, 1.5); *p* = 0.6	1.1 (0.7, 1.5); *p* = 0.8
**GDF-15 quartiles (ng/L)**				
Q1 (< 736)	68 (21.7%)	Ref	Ref	Ref
Q2 (736-971)	82 (26.0%)	1.3 (0.9, 1.8); *p* = 0.2	1.1 (0.7, 1.6); *p* = 0.8	1.0 (0.7, 1.5); *p* = 0.99
Q3 (1000-1426)	73 (23.3%)	1.1 (0.8, 1.6); *p* = 0.6	0.8 (0.5, 1.2); *p* = 0.2	0.8 (0.5, 1.2); *p* = 0.2
Q4 (> 1427)	92 (29.1%)	1.5 (1.0, 2.1); *p* = 0.03	0.9 (0.6, 1.4); *p* = 0.8	0.9 (0.6, 1.4); *p* = 0.6
ln hs-cTnT		1.37 (1.08, 1.74); *p* = 0.009	1.46 (1.09, 1.96); *p* = 0.01	1.7 (1.2, 2.4); *p* = 0.001
**hs-cTnT quartiles (ng/L)**				
Q1 (< 6)	79 (25.2%)	Ref	Ref	Ref
Q2 (6-8)	77 (24.4%)	1.0 (0.7, 1.4) *p* = 0.8	0.9 (0.6, 1.3); *p* = 0.6	1.0 (0.7, 1.5); *p* = 0.96
Q3 (8, 11)	59 (18.9%)	0.7 (0.5, 1.0); *p* = 0.06	0.7 (0.5, 1.0) *p* = 0.09	0.8 (0.5, 1.3); *p* = 0.4
Q4 (> 11)	100 (31.7%)	1.4 (1.0, 1.9); *p* = 0.08	1.4 (0.9, 2.1); *p* = 0.2	1.7 (1.1, 2.8); *p* = 0.03
	Demographics: age, gender, race/ethnicity (Black, White Chinese American, Hispanic)			
	Risk Factors: Hypertension, Diabetes, Lipids, Smoking, estimated glomerular filtration rate			
	Extra cellular volume, ECV; Growth Differentiation Factor-15, GDF-15; high sensitivity cardiac troponin T, hs-cTnT; Left ventricle, LV; Natural log, ln; Quartile, Q Odds ratios are reported as the OR (95% confidence intervals)			

## Discussion

In the present study using CMR we investigated the cross-sectional association of two pre-clinical imaging phenotypes of HF, replacement fibrosis and interstitial fibrosis, with hs-cTnT, a circulating cardiac-specific measure of myocyte injury, and GDF-15, a non-cardiac-specific biomarker considered an intermediary for inflammation, endothelial function and fibrosis (15). There are two key findings. First, for replacement fibrosis, as we have previously shown, there is a strong association between the presence of LGE and higher hs-cTn concentrations that exceeds a 7-fold odds ratio from the 1^st^ to 4^th^ quartile in adjusted models that include demographics, traditional CVD risk factors and LV mass (7). In contrast, and novel to this analysis, GDF-15 is associated with just a 2-fold higher prevalence of replacement fibrosis from the 1st to 4th quartile that is completely attenuated by adjustment for demographics and CVD risk factors. Second, neither biomarker has been evaluated in a CVD-free general population cohort for the presence and extent of interstitial fibrosis. Both biomarkers are only moderately associated with the CMR surrogate for interstitial fibrosis. The association of GDF-15 with interstitial fibrosis is also no longer present once adjusted for demographics. It is important to note that almost all the levels of GDF-15 and hs-cTnT reported in this study that are associated with subclinical replacement or interstitial fibrosis are within the reported normal range for general populations without known CVD (19, 21).

Release of cTn is a complex biochemical process and thought to be related due to cardiomyocyte cell death or injury. Whether low-concentration cTn release can solely be attributed to cardiomyocyte necrosis or even irreversible injury remains a subject of debate (22). A recent experimental study which investigated the temporal rise in hs-cTn during balloon coronary occlusions as short as 30 seconds in humans indicated that detection of cTn with an hs assay may not always be indicative of myocyte cell death (23). Replacement fibrosis, irrespective of its location within the myocardium, is generally considered irreversible irrespective of whether the mechanism of myocyte loss is secondary to necrosis or an alternative mechanism (16, 24, 25). Alternatively, increased ECV, the CMR surrogate for interstitial fibrosis, is potentially reversible as demonstrated by treatment with the sodium-glucose cotransporter-2 inhibitor empagliflozin in patients with HFrEF (26). It remains to be determined with both forms of fibrosis to what extent cTnT release is the result of myocyte cell death or reversible injury.

In contrast to the cardiac-specific nature of cTnT, GDF-15 is a circulating protein that plays a role in both cancers and cardiovascular diseases (27). Elevated concentrations are associated with apoptosis in solid tumors. However, GDF-15 is also known to be pleotropic with opposing effects that can lead to proliferation of cancer cells. Similar conflicting data have been found for GDF-15 in both promoting myocyte hypertrophy and protecting against it in animal models. GDF-15 effects also impact non-myocyte cardiac cells including endothelial cells and fibroblasts. Though not thought to be produced by cardiac fibroblasts, GDF-15 may promote activation and proliferation resulting in the progression of fibrosis (27). In human studies using targeted discovery proteomics and clustering in HFpEF patients, different findings have been reported. In a sub study of TOPCAT (Treatment of Preserved Cardiac Function Heart Failure with an Aldosterone Antagonist Trial), GDF-15 was labeled as an “intermediary metabolism” protein clustering with proteins associated with mineral metabolism including fibroblast growth factor-23 and osteoprotegerin (14). In contrast, in the PROMIS-HFpEF study GDF-15 was an exemplar protein in a cluster characterized by a primary pathway associated with leukocyte degranulation (28). Despite, or as a result of, the pleotropic roles suggested for GDF-15, it is consistently one of the most robust prognostic circulating protein biomarkers for predicting cardiovascular death and HF hospitalization in multiple populations including community dwelling individuals similar to participants in MESA (13, 29). Our findings show that progressive circulating GDF-15 concentrations, while associated with traditional CVD risk-factors and age, is not associated with the extent of interstitial fibrosis. This is also supported by our finding that GDF-15 is not associated with LV remodeling as measured on CMR by increasing LV end diastolic volume or mass. This contrasts with earlier findings in MESA showing a significant trend between progressive hs-cTnT levels and increasing LV mass (7). While GDF-15 is prognostic for incident HF and is strongly associated with HFpEF, its lack of association with CMR determined interstitial and replacement fibrosis suggests that it isn’t a marker of cardiac fibrosis in a pre-clinical general population cohort and is unlikely to have a causal role in the development of pre-clinical stage B cardiac fibrosis. This cross-sectional observation is further supported by the findings from EMPA-TROPISM, a mechanistic longitudinal clinical trial of non-diabetic patients with HFrEF treated with empagliflozin resulting in a decrease in interstitial fibrosis, but where GDF-15, one of the 92 circulating proteins studied, was not one of the 17 that significantly changed with treatment (26). Therefore, while GDF-15 was not associated with cardiac fibrosis in our study it is possible that other non-cardiac-specific circulating biomarkers such as soluble ST2, galectin-3, or procollagen III would be associated in a cross sectional analysis and some may have a causal role that warrants further investigation either as a single targeted biomarker analysis or as part of a proteomic discovery approach.

### Limitations

This is a cross-sectional analysis and as a result only associations between the circulating biomarkers and CMR measures of replacement and interstitial fibrosis can be identified; causality cannot be inferred. Gadolinium-based CMR was conducted approximately 10 years after study inception and participants with contraindications to gadolinium, including impaired renal function were excluded. Specific to this analysis, participants with interim clinical CVD events from study inception (*n* = 77) were also excluded, potentially limiting the generalizability of the results. Despite the relatively large number of subjects undergoing gadolinium enhanced CMR in MESA we were underpowered to look at effect modification with statistical interaction based on demographics and CVD risk factors. The prevalence of LGE is low as expected given the exclusion of individuals with known CVD from the study population. This results in reduced power to detect significant associations with the biomarkers and may be a factor in the absence of a statistically significant difference in the fully adjusted model with GDF-15 and LGE. To estimate the extent of interstitial fibrosis synthetic hematocrits needed to be used to calculate ECV, but previous studies have demonstrated that synthetic and measured hematocrit based ECV are highly correlated (20). ECV may also be sensitive to myocardial edema and inflammation, but such effects should be minimal because of the chronicity of alterations and the fact that it is a population of community dwellers, not patients. Additionally, our definition of interstitial fibrosis was somewhat arbitrary as the highest quartile since ECV is a continuous variable but is consistent with prior definitions used in MESA (30). Changing this definition could change the odds ratios but shouldn’t qualitatively impact the differences between hs-cTnT and GDF-15 with ECV. Both biomarkers were measured in samples that had been frozen for extended periods of time. However, robust measures of hs-cTnT have been shown in MESA at earlier time points with longer freeze times and GDF-15 has also been shown to have analytic stability with extended freeze times (7, 29).

## Conclusion

Hs-cTnT, a measure of ongoing myocyte injury or death, is strongly associated with CMR LGE estimated replacement and to a lesser extent interstitial fibrosis. In contrast, circulating GDF-15, a prognostically powerful pleiotropic protein associated with multiple adverse cardiovascular outcomes, is not independently associated with either CMR estimated replacement or interstitial fibrosis in stage B HF. These findings add mechanistic insights that could prove useful when considering using these biomarkers as entry criteria or surrogate endpoints for HF preventive cardiac-specific treatments.

## Data availability statement

The raw data will be made available by the authors or MESA as per data sharing agreements per MESA policies (https://www.mesa-nhlbi.org/default.aspx).

## Ethics statement

The studies involving human participants were reviewed and approved by Inova. Written informed consent for participation was not required for this study in accordance with the national legislation and the institutional requirements.

## Author contributions

CdF wrote the first draft of the manuscript, managed all editing, and took full responsibility for its contents. All authors have contributed with either writing or critically reviewed and edited this manuscript, and agreed with its contents. The manuscript has been approved by MESA for submission for publication.
