# Supplementary material for: Association of cardiac troponin T and growth differentiation factor 15 with replacement and interstitial cardiac fibrosis in community dwelling adults: The multi-ethnic study of atherosclerosis
Source: Front Cardiovasc Med. 2023 Feb 9;10:1104715. doi: 10.3389/fcvm.2023.1104715 (PMC9949377; doi:10.3389/fcvm.2023.1104715)
Supplement: Supplementary file 1 [file Data_Sheet_1.pdf]

Supplemental Material

Supplemental Tables 1a-1b.

Supplemental Tables 2a-2b.

Supplemental Table 3.

Supplemental Tables

Supplemental Table 1a. Patient Characteristics, by GDF-15 quartile (ng/L), among those with complete LGE measures without incident CVD (n=1737).

	**Quartile 1** (<738 ng/L)	**Quartile 2** (738-973 ng/L)	**Quartile 3** (973-1347 ng/L)	**Quartile 4** (≥1348 ng/L)	**p-value**
Age	61.7 (5.4)	65.8 (6.5)	71.1 (8.5)	73.1 (9.4)	<.001
Male	73 (46.2%)	81 (50.9%)	75 (47.8%)	86 (53.8%)	0.3
Race					
Caucasian	187 (43.1%)	185 (42.6%)	185 (42.5%)	218 (50.1%)	0.2
Chinese American	41 (9.5%)	40 (9.2%)	52 (12.0%)	31 (7.1%)	
Black	109 (25.1%)	119 (27.4%)	106 (24.4%)	98 (22.5%)	
Hispanic	97 (22.5%)	90 (20.7%)	91 (21.0%)	88 (20.2%)	
Hypertension	73 (46.2%)	82 (51.6%)	103 (65.6%)	114 (71.3%)	<.001
Diabetes	8 (5.1%)	20 (12.6%)	21 (13.4%)	59 (36.9%)	<.001
Smoking					
Never	219 (50.8%)	190 (43.8%)	185 (42.8%)	160 (37.0%)	.001
Former	190 (44.1%)	200 (46.1%)	215 (49.5%)	229 (52.9%)	
Current	22 (5.1%)	44 (10.1%)	33 (7.6%)	44 (10.2%)	
HDL-C	55.6 (16.7)	53.5 (15.9)	54.7 (16.7)	51.9 (13.2)	.06
LDL-C	110. 1 (29.2)	109.9 (27.5)	103.2 (31.2)	94.4 (34.2)	<.001
Total Chol	187.2 (35.6)	185.3 (32.4)	180.6 (36.3)	168.5 (38.3)	<.001
BMI (kg/m2)	28.4 (4.9)	28.5 (5.1)	28.2 (5.1)	28.4 (5.6)	0.8
eGFR (ml/min/1.73m^2)	78.6 (11.3)	74.7 (12.3)	71.0 (11.8)	66.1 (13.9)	<.001

SBP (mm Hg)	117.9 (18.2)	122.2 (20.0)	124.4 (18.7)	124.7 (19.1)	<.001
DBP (mm Hg)	69.4 (10.4)	70.4 (9.9)	69.1 (9.1)	66.9 (9.2)	<.001
Prevalent LGE	11 (2.5%)	24 (5.5%)	38 (8.8%)	39 (9.0%)	<.001
LVEF (%)	62.6 (6.5)	62.2 (6.4)	61.6 (7.3)	61.6 (7.1)	0.07
LVEDVI	67.8 (12.4)	66.3 (13.8)	66.3 (13.6)	63.0 (13.6)	<.001
LV mass (g)					
Male	148.6 (30.6)	150.0 (30.2)	144.4 (30.0)	141.1(27.3)	0.002
Female	103.9 (19.8)	105.1 (20.8)	104.8 (23.1)	105.1 (23.2)	0.6
Blood pressure, BP; Body mass index, BMI; Cardiac magnetic resonance, CMR; Estimated glomerular filtration rate, eGFR; Extra cellular volume, ECV; High density lipoprotein cholesterol; HDL-C; Hypertension, HTN; Low density lipoprotein cholesterol, LDL-C; Left ventricular diastolic volume index, LVEDVI; Left ventricular ejection fraction, LVEF.					

Supplemental Table 1b. Patient Characteristics, by GDF-15 quartile (ng/L), among those with complete ECV measures and without incident CVD (N=1258).

	**Quartile 1** (<736 ng/L)	**Quartile 2** (736-971 ng/L)	**Quartile 3** (972-1341 ng/L)	Quartile 4 (≥1342l ng/L)	**p-value**
Age	61.2 (5.5)	65.4 (6.8)	70.3 (8.0)	72.6 (9.2)	<.001
Male	151 (48.1%)	154 (48.9%)	169 (54.0%)	170 (53.8%)	.08
Race					0.3
Caucasian	155 (59.4%)	157 (49.8%)	150 (47.9%)	184 (58.2%)	
Chinese American	39 (12.4%)	36 (11.1%)	43 (13.7%)	26 (8.2%)	
Black	71 (22.6%)	79 (25.1%)	76 (24.3%)	63 (19.9%)	
Hispanic	49 (15.6%)	44 (14.0%)	44 (14.1%)	43 (13.6%)	
Hypertension	127 (40.5%)	147 (46.7%)	182 (58.2%)	205 (64.9%)	<.001
Diabetes	18 (5.7%)	36 (11.4%)	41 (13.1%)	98 (31.0%)	<.001
Smoking					
Never	156 (50.0%)	138 (43.8%)	129 (41.5%)	117 (37.0%)	0.02
Former	142 (45.5%)	143 (45.5%)	162 (52.1%)	165 (52.2%)	
Current	14(4.5%)	34 (10.8%)	20 (6.4%)	34 (10.8%)	
HDL-C	55.2 (15.7)	55.4 (16.4)	54.8 (16.1)	53.7 (16.4)	.15
LDL-C	114. 3 (29.2)	110.4 (27.3)	104.3 (30.9)	95.5 (32.3)	<.001
Triglycerides	97 [73, 139]	92 [68, 131]	98 [70, 141]	95 [70, 133]	0.6
BMI (kg/m2)	28.3 (5.1)	28.3 (5.2)	28.3 (5.1)	28.5 (5.8)	0.7
eGFR (ml/min/1.73m^2)	78.5 (11.2)	75.2 (12.2)	70.9 (12.2)	66.6 (13.7)	<.001
SBP (mm Hg)	117.3 (18.2)	120.6 (19.7)	123.8 (18.4)	124.3 (19.0)	<.001
DBP (mm Hg)	69.2 (10.6)	69.2 (9.6)	69.9 (9.3)	66.9 (9.1)	0.002
ECV %	26.3 (2.6)	26.8 (2.7)	26.7 (2.8)	27.1 (2.8)	0.6
LVEF (%)	62.6 (6.5)	62.2 (6.4)	61.6 (7.3)	61.6 (7.1)	0.03
LVEDVI	67.8 (12.7)	66.0 (12.9)	64.9 (13.6)	62.8 (13.9)	<.001
LV mass (g)					

Male	149.7 (32.6)	147.3 (29.0)	143.8 (29.0)	141.7 (27.0)	0.008
Female	101.7 (19.0)	103.5 (21.3)	102.8 (22.5)	102.7 (22.0)	0.8
Abbreviation as per supplemental Table 1a					

Supplemental Table 2a. Patient Characteristics, by hs-cTnT quartile (ng/L), among those with complete LGE measures without incident CVD (n=1737).

	**Quartile 1** (<5.9 ng/L)	**Quartile 2** (5.9-8.0 ng/L)	**Quartile 3** (8.1-11.2 ng/L)	**Quartile 4** (>11.2 ng/L)	**p-value**
Age	62.1 (5.8)	65.8 (7.3)	68.9 (8.4)	73.5 (9.1)	<.001
Male	112 (25.9%)	195 (45.0%)	264 (60.7%)	315 (72.3%)	<.001
Race					<.001
Caucasian	178 (41.1%)	210 (48.5%)	174 (40.0%)	213 (48.9%)	
Chinese American	65 (15.0%)	34 (7.9%)	41 (9.4%)	24 (5.5%)	
Black	85 (19.6%)	100 (23.1%)	120 (27.6%)	127 (29.1%)	
Hispanic	105 (24.3%)	89 (20.6%)	100 (23.0%)	72 (16.5%)	
Hypertension	152 (35.1%)	219 (50.6%)	254 (58.4%)	307 (70.4%)	<.001
Diabetes	41 (9.5%)	48 (11.1%)	83 (19.1%)	106 (24.3%)	<.001
Smoking					<.001
Never	223 (51.6%)	185 (42.7%)	177 (40.9%)	169 (39.1%)	
Former	171 (39.6%)	209 (48.3%)	215 (49.7%)	238 (55.1%)	
Current	38 (8.8%)	39 (9.0%)	41 (9.5%)	25 (5.8%)	
HDL-C	56.7 (15.9)	55.8 (15.9)	53.1 (16.5)	53.5 (16.1)	0.8
LDL-C	115.8 (2.9)	108.1 (30.8)	106.9 (32.7)	97.6 (30.4)	<.001
Total Chol	195.4 (32.1)	185.1 (35.4)	182.1 (36.8)	172.5 (35.8)	.03
BMI (kg/m2)	27.7 (4.9)	28.6 (5.6)	28.7 (5.0)	2.5 (5.2)	.02
eGFR (ml/min/1.73m^2)	78.5 (11.8)	73.8 (12.1)	71.1 (12.3)	66.9 (13.8)	.004
SBP (mm Hg)	116.7 (17.4)	121.5 (17.9)	124.5 (19.8)	126.4 (20.3)	.002
DBP (mm Hg)	68.2 (9.3)	69.0 (10.3)	69.8 (9.4)	68.9 (9.9)	0.2

Prevalent LGE	3 (0.7%)	17 (3.9%)	27 (6.2%)	65 (14.9%)	<.001
LVEF (%)	62.8 (6.1)	62.0 (6.7)	61.6 (6.9)	60.1 (7.5)	.08
LVEDVI	64.4 (11.1)	65.9 (12.6)	65.8 (14.7)	66.7 (15.0)	.06
LV mass (g)					
Male	135.5 (23.2)	144.9 (25.4)	144.9 (28.5)	150.9 (33.8)	<.001
Female	99.9 (18.5)	105.7 (21.8)	107.0 (20.8)	112.2 (27.0)	<.001

Blood pressure, BP; Body mass index, BMI; Cardiac magnetic resonance, CMR; Estimated glomerular filtration rate, eGFR; Extra cellular volume, ECV; High density lipoprotein cholesterol; HDL-C; Hypertension, HTN; Low density lipoprotein cholesterol, LDL-C; Left ventricular diastolic volume index, LVEDVI; Left ventricular ejection fraction, LVEF.

Supplemental Table 2b. Patient Characteristics, by hs-cTnT quartile (ng/L), among those with complete ECV measures without incident CVD (N=1258).

	**Quartile 1** (<5.84 ng/L)	**Quartile 2** (5.84-8.02 ng/L)	**Quartile 3** 8.03-11.22 ng/L)	**Quartile 4** >11.22 ng/L	**p-value**
Age	62.3 (6.0)	65.8 (7.4)	68.5 (8.3)	72.9 (9.2)	<.001
Male	78 (24.9%)	136 (43.0%)	198 (63.3%)	232 (73.4%)	<.001
Race					<.001
Caucasian	147 (47.0%)	171 (54.1%)	149 (47.6%)	179 (56.7%)	
Chinese American	59 (18.9%)	31 (9.8%)	32 (10.2%)	21 (6.7%)	
Black	57 (18.2%)	68 (21.5%)	83 (26.5%)	81 (25.6%)	
Hispanic	50 (16.0%)	46 (14.6%)	49 (15.7%)	35 (11.1%)	
Hypertension	110 (35.1%)	157 (49.7%)	180 (57.5%)	214 (67.7%)	<.001
Diabetes	827 (8.6%)	36 (11.4%)	56 (17.9%)	74 (23.4%)	<.001
Smoking					<.001
Never	158 (50.6%)	132 (41.8%)	129 (41.2%)	121 (38.7%)	
Former	126 (40.4%)	157 (49.7%)	158 (50.5%)	171 (54.6%)	
Current	28 (9.0%)	27 (8.5%)	26 (8.3%)	21 (6.7%)	
HDL-C	47 (16.5)	55.4 (15.6)	52.3 (15.2)	52.6 (15.7)	0.5
LDL-C	115.6 (28.4)	106.4 (28.8)	106.7 (32.1)	96.0 (29.5)	0.2
Total Chol	195.8 (31.8)	183.3 (34.9)	181.3 (36.1)	170.8 (34.6)	0.1
BMI (kg/m2)	27.5 (5.0)	28.5 (5.7)	28.7 (4.8)	28.8 (5.4)	.01
eGFR (ml/min/1.73m^2)	78.7 (11.5)	73.2 (12.4)	71.2 (12.1)	68.1 (14.0)	.004
SBP (mm Hg)	116.5 (18.3)	119.5 (17.0)	124.3 (19.4)	125.6 (20.0)	.02
DBP (mm Hg)	67.6 (9.5)	68.1 (10.0)	69.8 (9.4)	68.9 (9.9)	0.7

ECV %	26.6 (2.6)	26.8 (2.7)	26.4 (2.6)	27.1 (3.1)	.007
LVEF (%)	63.4 (6.1)	62.4 (6.7)	61.8 (6.8)	60.4 (7.3)	.01
LVEDVI	63.8 (11.0)	63.4 (12.6)	65.8 (14.3)	66.5 (15.2)	<.001
LV mass (g)					
Male	133.5 (22.8)	144.6 (26.0)	145.1 (27.9)	150.3 (33.2)	<.001
Female	98.0 (18.4)	104.3 (21.8)	104.5 (19.0)	109.5 (26.4)	<.001
Blood pressure, BP; Body mass index, BMI; Cardiac magnetic resonance, CMR; Estimated glomerular filtration rate, eGFR; Extra cellular volume, ECV; High density lipoprotein cholesterol; HDL-C; Hypertension, HTN; Low density lipoprotein cholesterol, LDL-C; Left ventricular diastolic volume index, LVEDVI; Left ventricular ejection fraction, LVEF.					

Supplemental Table 3: Association of GDF-15 and hs-cTnT with % ECV (continuous outcome variable).

	**Unadjusted**	**Demographic Adjusted**	**Demographic + Risk Factor + LV Mass Adjusted**
ln GDF-15	0.73 (0.41, 1.06)<br>P<.001	0.23 (-0.12, 0.59)<br>p=0.2	0.19 (-0.21, 0.59)<br>p=0.3
GDF-15 Quartiles (ng/L)			
Q1 (<736)	Ref	Ref	Ref
Q2 (736-971)	0.44 (0.00, 0.87)<br>p=0.2	0.13 (-0.28, 0.56)<br>p=0.5	0.07 (-0.34, 0.49)<br>p=0.7
Q3 (1000-1426)	0.42 (-.01, 0.85)<br>p=0.6	-0.15 (-0.60, 0.30)<br>p=0.5	-0.17, (-0.62, 0.23)<br>p=0.5
Q4 (>1427)	0.82 (0.39, 1.25)<br>p=0.03	0.09 (-0.37. 0.56)<br>p=0.7	0.03, (-0.47, 0.53)<br>p=0.9
ln hs-cTnT (per 1-ln unit)	0.45 (0.16, 0.74)<br>p=.002	0.46 (0.13, 0.79)<br>p=.008	0.60 (0.25, 0.95)<br>p=.001
hs-cTnT Quartiles (ng/L)			
Q1 (<6)	Ref	Ref	Ref
Q2 (6-8)	0.15 (-0.28, 0.58)<br>p=0.4	0.13 (-0.29, 0.55)<br>p=0.5	0.24 (-0.19, 0.67)<br>p=0.3
Q3 (8, 11)	-0.23 (-0.67, 0.19)<br>p=0.4	-0.19 (-0.63, 0.27)<br>p=0.4	-0.01 (-0.47, 0.46)<br>p=0.9
Q4 (>11)	0.48 (0.05, 0.91)<br>p=0.2	0.37 (-0.13, 0.87)<br>p=0.1	0.57 (0.04, 1.09)<br>p=.04
Cell values represent beta-coefficients from linear regression models and 95% confidence intervals			
Demographics: age, gender, race/ethnicity (Black, White Chinese American, Hispanic)			
Risk Factors: Hypertension, Diabetes, Lipids, Smoking, estimated glomerular filtration rate			

Extra cellular volume, ECV; Growth Differentiation Factor-15, GDF-15; high sensitivity cardiac troponin T, hs-cTnT; Left ventricle, LV; Natural log, ln; Quartile, Q
